# Organelles and cancer cell pyroptosis: overview and perspectives

**DOI:** 10.1038/s41419-025-08371-9

**Published:** 2025-12-27

**Authors:** AnPeng Qiu, JunDa Lin, HaoRan Hu, ZiHou Zhao, XinTong Cai, Yuyue Zhao, GuangTao Yu

**Affiliations:** 1 Stomatological Hospital, School of Stomatology, Southern Medical University, Guangzhou Guangdong, 510280 China; 2 The Eighth Affiliated Hospital, Southern Medical University (The First People’s Hospital of Shunde, Foshan), Foshan, 528300 China

**Keywords:** Cancer immunotherapy, Cell death

## Abstract

Pyroptosis, a gasdermin (GSDM)-mediated immunogenic programmed cell death modality, manifests through characteristic membrane permeabilization and proinflammatory cytokine release. Pyroptosis exhibits dual therapeutic advantages by remodeling the tumor microenvironment and potentiating systemic anti-tumor immunity, positioning it as a pivotal focus in cancer immunotherapy. However, researchers still focus current pyroptosis induction strategies predominantly on single molecular targets and have not sufficiently analyzed the inter-organelle communication networks that govern pyroptotic signaling cascades. This review provides a systematic exploration of organelle-specific ultrastructural alterations during pyroptosis progression and the molecular machinery regulating organelle-mediated pyroptotic pathways. We synthesize recent advances in organelle-targeted pyroptosis induction strategies, elucidating how inter-organelle crosstalk networks to enhance therapeutic efficacy. We aim to provide translational approaches for optimizing cancer treatment paradigms.

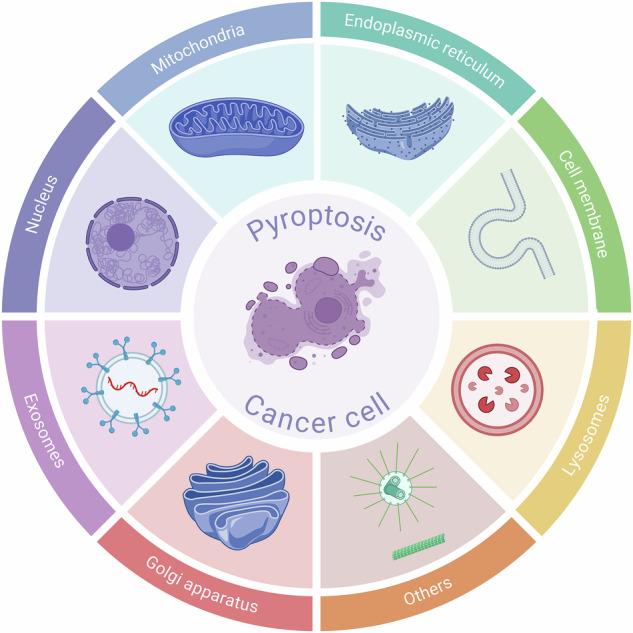

## Facts

- Organelle-centric pyroptosis regulation: Organelles (nucleus, mitochondria, lysosomes, Golgi, endoplasmic reticulum (ER), cytoskeleton) coordinate pyroptosis via transcriptional control, reactive oxygen species (ROS)/mitochondrial DNA (mtDNA) release, cathepsin activation, palmitoylation, unfolded protein response (UPR) signaling, and inflammasome scaffolding, defining spatiotemporal execution of immunogenic cell death.
- Membrane dynamics as therapeutic targets: GSDM pore formation, endosomal sorting complex required for transport (ESCRT)-mediated repair, and ninjurin-1 (NINJ1)-driven rupture offer leverage points for nanomaterial/photodynamic therapies to induce pyroptosis while evading membrane repair mechanisms in tumor cells.
- Inter-organelle crosstalk amplifies pyroptosis: Mitochondrial-lysosomal Ca²⁺/ROS exchange, nuclear-mitochondrial sirtuin 1 (SIRT1)/nuclear factor-kappa B (NF-κB) signaling, and ER-Golgi vesicular trafficking create self-reinforcing death loops that remodel tumor microenvironments and potentiate systemic immunity.

## Open Questions

- How can organelle-specific damage (e.g., lysosomal permeabilization, mitochondrial cristae loss) be quantitatively standardized as biomarkers to stratify pyroptosis efficacy across heterogeneous tumor types?
- What strategies can selectively induce tumor pyroptosis via organelle targeting (e.g., Golgi-disrupting agents, ER-phagy inducers) while avoiding uncontrolled systemic inflammation from cytokine/damage-associated molecular patterns (DAMP) release?
- Can real-time tracking of organelle dynamics (e.g., mtDNA release, vimentin cleavage) predict therapeutic resistance and enable dynamic adjustment of pyroptosis-focused regimens?

## Introduction

Targeted regulation of programmed cell death mechanisms has emerged as a paradigm in cancer therapeutics. Traditional apoptosis-based therapies have long been constrained by tumor cell p53 mutations, apoptotic resistance, and microenvironment-mediated immune evasion [1, 2]. Pyroptosis, characterized by plasma membrane perforation through GSDM protein family, releases proinflammatory cytokines (e.g., IL-1β, IL-18) while directly eliminating tumor cells and activating antitumor immunity, demonstrating superior “immunogenic cell death” characteristics compared to apoptosis [3, 4]. The elucidation of GSDMD as the pyroptosis executioner has accelerated research in this field [5]. Targeting pyroptosis offers new avenues to overcome therapeutic bottlenecks in tumors.

Inflammasome-activated caspase-1/4/5/11 execute pyroptosis, which cleave GSDM protein into an N-terminal fragment (GSDM-N) and a C-terminal fragment (GSDM-C). The liberated GSDM-N oligomerizes to form nanoscale membrane pores, inducing osmotic lysis and DAMP release. [6]. This unique death modality activates dendritic cells and cytotoxic T lymphocytes to remodel immunosuppressive microenvironments, demonstrating significant tumor regression in preclinical studies [7]. However, pyroptosis induction therapy faces three core challenges: (1) Uncontrolled systemic inflammation potentially triggering cytokine storms; (2) Tumor heterogeneity causing GSDM expression variations affecting efficacy; 3) Emergence of pyroptosis escape mechanisms [8].

Deeper limitations stem from insufficient understanding of subcellular organelle crosstalk in dynamic regulatory networks. For instance, mitochondria participate in pyroptosis initiation through ROS modulation and mtDNA release, while endoplasmic reticulum (ER) stress regulates GSDMD expression via protein kinase R-like endoplasmic reticulum kinase (PERK) / eukaryotic translation initiation factor 2 subunit alpha (eIF2α) pathways, and lysosomal membrane permeabilization (LMP) activates non-canonical caspase-1 pathways, revealing sophisticated crosstalk among these organelles [9–11]. Particularly, Mitochondrial-lysosomal crosstalk highlights the critical role of spatial reorganization in death signal transduction [12, 13]. This review systematically elucidates pyroptosis from a subcellular organelle dynamics perspective. We specifically discuss organelle-specific biological responses and dynamic crosstalk networks in pyroptotic processes, not only advancing mechanistic understanding, but also mapping routes for translating organelle-centric strategies into precision oncology and immunomodulation.

## Mechanistic overview of pyroptosis

The mechanisms of pyroptosis are generally grouped into four major categories: the canonical, non-canonical, apoptosis-induced, and granzyme pathways. In the canonical pathway, inflammasomes such as NOD-like receptor family pyrin domain-containing 3 (NLRP3) activate caspase-1 to process both GSDMD and proinflammatory cytokines. By contrast, in the non-canonical pathway, cytosolic lipopolysaccharide (LPS) engages caspase-4/5/11, which cleave GSDMD and secondarily activate NLRP3/caspase-1 via K⁺ efflux. Meanwhile, apoptotic caspases can redirect cell death toward pyroptosis, as caspase-3 cleaves GSDME during chemotherapy, whereas caspase-8 and others target additional GSDM under stress conditions. Notably, cytotoxic lymphocytes provide an immune axis of regulation, since granzyme B and A cleave GSDME and GSDMB, respectively, thereby inducing pyroptosis independently of caspases [14]. Beyond these established routes, emerging mechanisms such as alternative proteases, GSDM lipid modifications, and metabolic cues further expand regulatory diversity [15–17]. Taken together, these interconnected pathways converge on GSDM activation and pore formation, ensuring redundancy in host defense while offering multiple therapeutic opportunities in cancer.

## Nucleus

### Nuclear proteome dynamics: mechanistic alterations in core regulatory protein

**Fig. 1:**
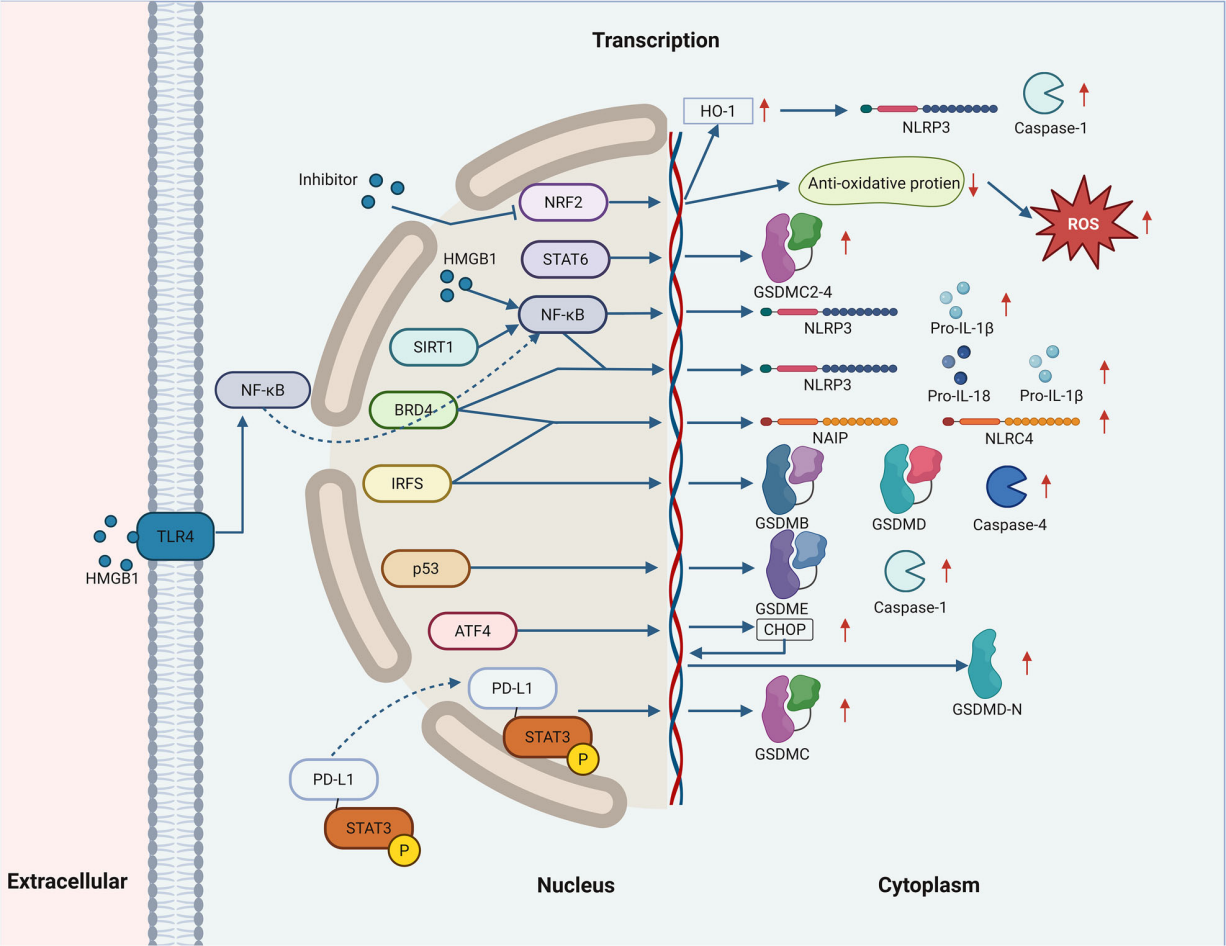
Nuclear regulatory mechanisms driving tumor cell pyroptosis through transcriptional control of pyroptosis-associated proteins. Extracellular HMGB1 activates TLRs to promote NF-κB translocation and DNA binding. SIRT1 sustains NF-κB expression in neoplastic cells (MCF-7/H1299). BRD4 synergizes with NF-κB to transcribe pro–IL-1β and NLRP3 and partners with IRF8 to expand neuronal apoptosis inhibitor protein (NAIP)/NLR family CARD domain-containing protein 4 (NLRC4) inflammasome diversity. IRF1 compensates for IRF2 deficiency in regulating GSDMD expression; IRF1 promotes AIM2 during Francisella infection, whereas IRF2 preferentially induces caspase-4, and both may enhance GSDMB expression. p53 directly activates caspase-1 and GSDME transcription. ATF4 upregulates CHOP, which subsequently drives caspase-1–mediated GSDMD cleavage. Under hypoxia, nuclear PD-L1 associates with phosphorylated STAT3 (Y705) to form a transcriptional complex that engages STAT3 response elements within the GSDMC promoter. Collectively, NRF2, STAT6, NF-κB, HMGB1, SIRT1, BRD4, IRF5, p53, and ATF4, collaboratively regulate the transcription of pyroptosis-related executors and inflammasome components. These include GSDM family members (GSDMD, GSDMC, GSDMB, GSDME), caspases (caspase-1, caspase-4), pro-inflammatory cytokine precursors (Pro-IL-1β, Pro-IL-18), and inflammasome sensors (NLRP3, NAIP, NLRC4). These molecular events orchestrate pyroptosis through membrane pore formation and inflammatory cascade activation.

Transcriptional orchestration of pyroptosis constitutes a sophisticated regulatory network spanning redox homeostasis, inflammatory signaling, and oncogenic pathways. Among these regulators, Nuclear factor erythroid 2-related factor 2 (NRF2) acts as a brake by limiting intracellular ROS accumulation and thereby suppressing NLRP3/caspase-1 activation through maintenance of antioxidant defenses [18–22]. In contrast, Signal Transducer and Activator of Transcription 6 (STAT6) couples immune signaling with cell death by linking IL-4 responsiveness to the transcriptional activation of GSDMC isoforms [23]. At the center of inflammatory circuits, NF-κB functions as a pivotal hub that directly induces inflammasome components such as NLRP3 and pro-IL-1β, while its activity is further fine-tuned by upstream modulators including high mobility group box 1 (HMGB1), SIRT1, and bromodomain-containing protein 4 (BRD4) [24–28]. Complementary control is provided by the interferon regulatory factor (IRF) family, in which IRF1 and IRF2 differentially regulate GSDMD, absent in melanoma 2 (AIM2), and caspase-4, thereby ensuring transcriptional flexibility in response to diverse stimuli [23, 29–31]. In oncological contexts, p53 bridges genomic surveillance with pyroptosis by transcriptionally activating caspase-1 and GSDME, whereas under hypoxic stress, nuclear translocation of programmed death-ligand 1 (PD-L1) together with phosphorylated signal transducer and activator of transcription 3 (STAT3) forms a transcriptional complex that selectively drives GSDMC expression [32–34]. Moreover, the activating transcription factor 4 (ATF4)/C/EBP-homologous protein (CHOP) axis contributes to caspase-1-dependent activation of GSDMD during endoplasmic reticulum stress [10, 35]. Collectively, these transcriptional programs establish a dynamic regulatory framework through which tumor cells integrate microenvironmental cues into pyroptotic sensitivity, highlighting potential therapeutic avenues ranging from redox modulation to interception of inflammatory circuits and exploitation of oncogenic stress pathways (Fig. 1).

### Morphological changes of nucleus in pyroptosis

Morphological hallmarks of nuclear alterations during pyroptotic progression were quantitatively characterized under electrochemical stimulation. Distinct nuclear remodeling was observed, featuring progressive nuclear condensation accompanied by nucleolar fusion events, manifested as irregular nucleolar morphology and quantifiable reduction in nucleolar count [36]. These structural changes align with canonical pyroptotic biomarkers while sharing apoptotic features like chromatin fragmentation (TUNEL + ) and phosphatidylserine exposure (Annexin V + ), pyroptotic nuclei specifically lack the 160–200 bp DNA laddering pattern characteristic of classical apoptosis, suggesting differential endonuclease activation patterns during inflammatory cell death [34].

Emerging therapeutic strategies targeting nuclear regulators of pyroptosis demonstrate significant potential for multimodal cancer intervention. This systematic synthesis (Table 1) delineates molecular circuitry linking nuclear targets to pyroptotic execution, detailing their mechanistic pathways, pharmacological relationships, and tumor-specific contexts. Key findings reveal that nuclear proteins—including NF-κB, BRD4, SIRT1, ATF4, NRF2, and HMGB1—orchestrate pyroptosis through transcriptional, epigenetic, and stress-response axes across diverse malignancies.

**Table 1:** The regulatory mechanism of pyroptosis by nuclear proteins.

Target	Mechanism	Cancer Model	Cell lines	Ref.
NF-κB	NF-κB/Caspase-3(8)/GSDME axis activation	Human gastric cancer	BGC823/DDP, SGC7901/DDP	[38]
	NF-κB/NLRP3/Caspase-1/GSDMD pathway activation	NSCLC	H1703, PC9	[25]
BRD4	BRD4-NLRP3/GSDMD complex potentiation	Colorectal cancer	SW620, LS174T, HCT116, DLD1	[44]
SIRT1	AMPK/SIRT1/NF-κB signaling axis amplification	General tumor models	HepG2, MCF-7, HCT-15, HT-29	[26]
ATF4	PERK/ATF4-dependent pyroptotic activation	Oral squamous cell carcinoma	CAL27, SCC-7	[10]
NRF2	NRF2/ROS/TXNIP/NLRP3 cascade initiation	Esophageal squamous carcinoma	KYSE-30	[19]
	NRF2 nuclear exclusion → NLRP3/caspase-1↑	Colorectal cancer	HT29	[21]
HMGB1	HMGB1/RAGE pathway-mediated pyroptosis	Lung cancer	A549, H1299	[45]

Arrows indicate mechanistic relationships (→ activation/induction). Upward arrows (↑) denote increased expression/activity.

### Anti-cancer therapy of nuclear factors

The dual regulatory role of NF-κB in tumor pyroptosis presents both therapeutic opportunities and mechanistic complexities. On the one hand, NF-κB activation promotes pyroptosis: Carvedilol facilitates NLRP3/apoptosis-associated speck-like protein containing a CARD (ASC)/caspase-1 assembly in prostate cancer [37], Tanshinone I triggers the NF-κB/caspase-3(8)/GSDME axis in cisplatin-resistant gastric cancer [38], and Polyphyllin VI induces GSDMD-dependent pyroptosis in non-small cell lung cancer (NSCLC) via NF-κB/NLRP3/caspase-1 signaling [39]. Conversely, NF-κB inhibition can also enhance pyroptosis, as shown by dopamine receptor D2-mediated NF-κB suppression in breast cancer. [25], Tanshinone IIinduced pyroptosis in cervical cancer [25], and ROS-dependent NF-κB blockade by the piperlongumine analog L50377 in NSCLC [39]. These paradoxical outcomes reflect the context-dependent nature of NF-κB transcriptional programs. Under chronic or low-grade inflammation, NF-κB predominantly drives anti-apoptotic genes (e.g., Bcl-2, XIAP), thereby attenuating caspase activation and suppressing pyroptosis [40, 41]. In contrast, acute or excessive stress such as ROS burst, oxidative damage, or metabolic imbalance can redirect NF-κB activity toward pro-pyroptotic functions, notably the induction of inflammasome components (NLRP3, pro-IL-1β) and GSDM (GSDMD, GSDMB) [42]. For example, in lung epithelial cells, zinc oxide nanoparticle-induced ROS activates NF-κB-dependent NLRP3 transcription, which is essential for subsequent pyroptosis and can be blocked by ROS scavengers or NF-κB inhibition [43]. Collectively, microenvironmental stress intensity emerges as a key determinant biasing NF-κB toward either survival or pyroptotic outputs, although the precise molecular switches remain unclear.

Beyond NF-κB, several other regulators have therapeutic implications. BRD4 acts as an epigenetic enhancer of pyroptosis, with histone deacetylase 2 inhibition amplifying BRD4-driven NLRP3/GSDMD activation in colorectal cancer [44]. SIRT1 exerts context-specific control, where metformin-induced AMP-activated protein kinase (AMPK)/SIRT1 signaling enhances NF-κB-primed pyroptotic cascades [26]. Nanomedicine approaches also harness stress-responsive pathways, exemplified by mannose-functionalized metal-organic frameworks that activate the PERK/ATF4 axis to elicit tumor-specific pyroptosis in vivo [10]. Redox modulation further illustrates dual targeting of the NRF2 pathway: piperlongumine promotes NRF2/ROS/ thioredoxin-interacting protein (TXNIP)/NLRP3-dependent pyroptosis in esophageal squamous carcinoma [19], whereas ginsenoside Rh3 impairs NRF2 nuclear translocation, enhancing NLRP3/caspase-1 activation in colorectal cancer [21]. Microenvironmental regulation through danger signal pathways is demonstrated by isoflurane-mediated activation of the HMGB1/receptor for advanced glycation end-products (RAGE) axis, which promotes pyroptosis in lung cancer systems [45].

## Membranes

### The pore-forming mechanism of GSDM in pyroptotic cell membranes

GSDM disrupts the cell membrane by pore formation during pyroptosis. The pore-forming mechanism of GSDM comprises five key steps: autoinhibition, proteolytic cleavage, conformational change, selective lipid binding and oligomerization, and pore formation.

The GSDM-N, rather than the full-length GSDM or the GSDM-C, is responsible for pyroptotic activity [46, 47]. The GSDM-C typically contains multiple hydrophobic residues that inhibit the pore-forming activity of the GSDM-N by forming a hydrophobic pocket that binds to the β1-β2 hairpins of the GSDM-N [48]. Caspases can activate GSDM not only through hydrolytic cleavage but also by processing the p22/p10 heterodimer to recruit GSDM [49–51]. Upon release from its self-inhibition state through hydrolysis, GSDMD-N undergoes a dramatic conformational change, forming an elongated and open β-hairpin loop that acts as a membrane-binding element and targets negatively charged membrane phospholipids [46, 48, 52, 53]. Selective binding of GSDMD-N to acidic phospholipids promotes the targeted aggregation of GSDMD to the cell membrane [46, 47]. The lateral interaction of the basic patches and β3-β8 in GSDMD promotes the GSDMD-N oligomerization, and the hydrophobic residues in the β1-β2 loop strengthen its interaction with the membrane, anchoring the complex [54]. There are two hypotheses of pore-formation: one in which the pore grows directly on the membrane without a “vertical collapse” phenomenon [54], and another in which the pre-pore transitions to a pore [55]. The exact mechanism of GSDMD pore assembly requires further investigation.

### Cellular molecular events initiated by GSDM pore formation in pyroptosis

GSDM pore formation in the cell membrane triggers a series of intracellular and extracellular changes. These changes involve alterations in the ion gradient, the selective release of cytokines and inflammatory mediators, and play a crucial role in pyroptosis and the inflammatory response.

The most immediate effect of GSDM pore formation on the cell membrane is the drastic disruption of ion flow, leading to the collapse of the ion gradient [56]. These pores, with their large internal diameters, allow uncontrolled K^+^ efflux and Ca^2+^ and Na^+^ influx, which in turn activate or inhibit various signal transduction pathways. K^+^ efflux promotes the NLRP3/caspase-1/GSDMD pathway [57], inhibits the cyclic GMP-AMP synthase-stimulator of interferon genes (cGAS-STING) pathway [58], and Ca^2+^ influx plays a role in phospholipid transformation and membrane repair [59].

Another significant function of the GSDMD pore is mediating the release of cytokines and inflammatory mediators. Key inflammatory factors such as IL-1β, IL-18, and IL-33 can be released through these pores without causing plasma membrane rupture (PMR) [60]. The molecular weight cutoff for passage through the GSDMD pore is ~40 kDa [46]. Small DAMPs like mature IL-1β, IL-18 (17/18 kDa), and galectin-1 (14 kDa) can diffuse across the GSDMD-permeabilized membrane [61–63], whereas larger DAMPs such as tetrameric lactate dehydrogenase (~140 kDa) and HMGB1 ( ~ 150 kDa) cannot [61, 62, 64]. This indicates that GSDMD pores primarily allow molecules of a certain size to pass, though alternative secretion routes may exist. However, mature IL-1β and IL-18, which are similar in size to their precursors, are released more rapidly [54], which cannot be explained solely by pore size limitation. The difference in surface charges of mature and precursor IL-1β (due to caspase removal of acidic patches in precursor IL-1β) and the acidic patches on the inner wall of the GSDMD pore may explain this anomaly [54]. These findings highlight the dual function of the GSDMD pore, which not only triggers pyroptosis but also serves as a selective, charge-specific channel for the non-lytic secretion of cytokines and inflammatory mediators.

### Membrane rupture and membrane repair mechanisms in pyroptosis

Although GSDMD pore formation during pyroptosis leads to water influx, osmotic swelling and membrane damage [65], the process of eventual PMR is regulated by additional mechanisms, particularly the action of NINJ1 [66]. Although the mechanism of NINJ1 activation remains unclear, oligomerization of NINJ1 and its α-helix structure are critical for its role in PMR. It is thought that the α1 and α2 helices recognize negatively charged phospholipids on the surface of pyroptotic cells [67].

Following GSDM pore formation and subsequent membrane damage, cells not only undergo cell lysis but also activate various membrane repair mechanisms to restore membrane integrity. The acid sphingomyelinase (ASM)-dependent endocytosis mechanism of GSDM pores and ESCRT-III-mediated impaired membrane shedding are essential for limiting cell death and regulating cytokine release [68–70].

During pyroptosis, GSDM pore formation causes Ca^2+^ influx, triggering lysosomal exocytosis. This results in the release of ASM-containing vesicles into the extracellular space at the site of membrane pore formation. The vesicles hydrolyze sphingolipids on the outer leaflets of the plasma membrane, producing ceramides that promote the formation of lipid rafts. This enhances local membrane invagination, which facilitates the removal of the GSDM pore by endocytosis [71–75]. Additionally, caspase-1 can cleave and activate caspase-7 [76], which crosses the GSDM pore and cleaves full-length ASM into a more active form, enhancing membrane repair [68].

The ESCRT machinery, consisting of ESCRT-I, ESCRT-II, ESCRT-III, apoptosis-linked gene-2 (ALG-2)-interacting protein X (ALIX), and vacuolar protein sorting-associated protein 4 (VPS4) complexes, is crucial for membrane repair [77]. Ca²⁺ binds to phosphatidylserine to recruit annexin A7 following plasma membrane injury [78], initiating membrane repair by recruiting ESCRT-related Ca^2+^-binding proteins like ALG-2 and ALIX [70, 79]. ESCRT-III components, such as charged multivesicular body protein 4B, are recruited to the site of membrane injury [69, 80]. Although the exact mechanism of ESCRT-III-induced membrane shedding remains unclear, ESCRT-III may form coiled, spring-like polymers that apply mechanical buckling forces on the lipid membrane, leading to vesicle shedding [81, 82]. VPS4 is responsible for recycling ESCRT-III after membrane detachment [69]. ensuring that the ESCRT machinery is available for subsequent rounds of repair [77].

**Fig. 2:**
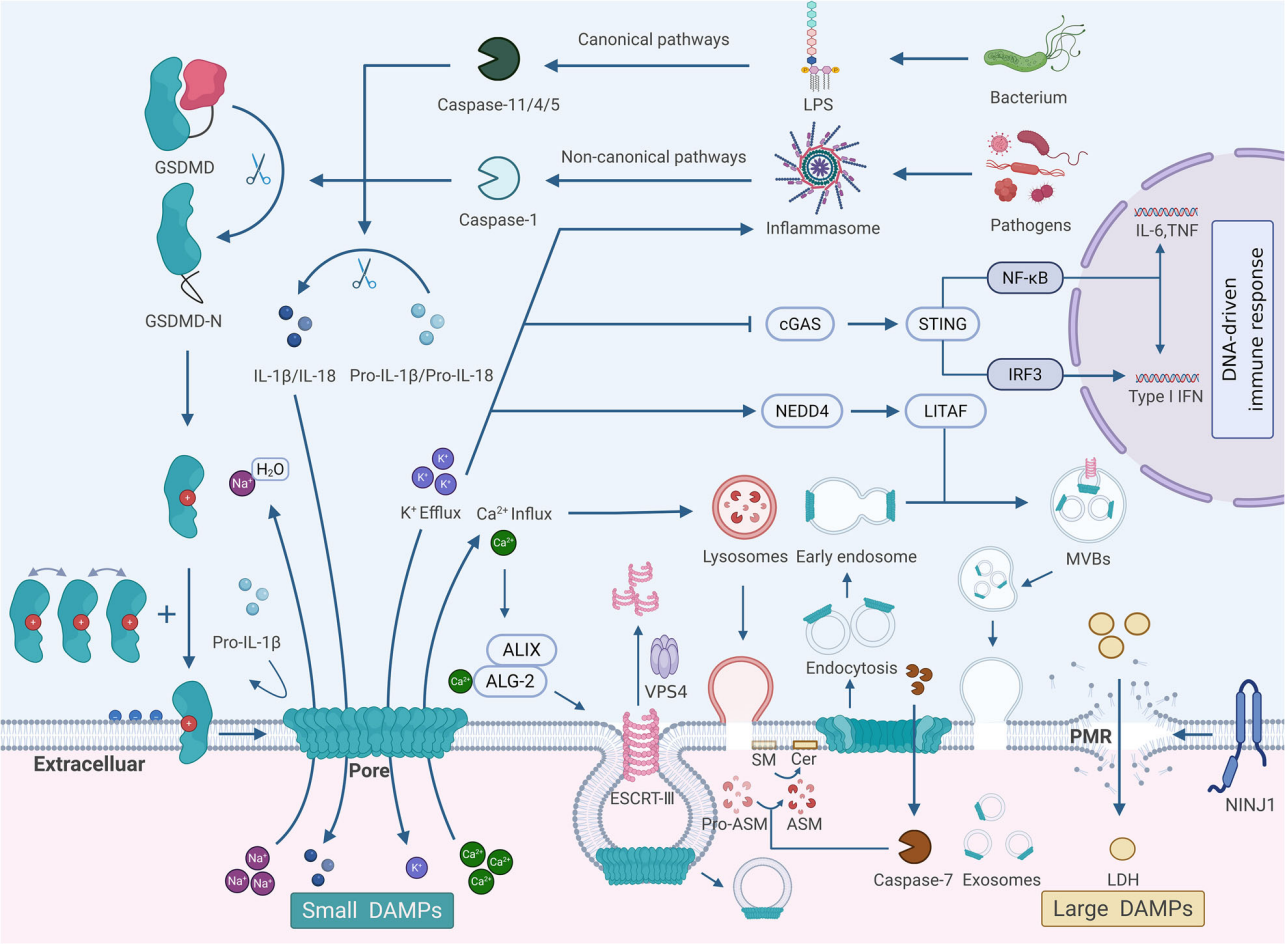
Pore formation, membrane rupture, membrane repair mechanisms, and the coordination of related molecular cascades in pyroptotic cell membranes. Canonical inflammasome activation triggers caspase-1, while caspase-11/4/5 engage via cytosolic LPS detection. Caspase-mediated GSDMD cleavage liberates its pore-forming GSDMD-N, concurrently maturing IL-1β/IL-18. GSDMD-N oligomerizes on anionic membranes, creating pores that mediate ionic/small-molecule flux while retaining pro-IL-1β/pro-IL-18. K^+^ efflux activates NLRP3, inhibits the immune response to cytosolic DNA and IRF3-mediated interferon production in the cGAS-STING pathway, and mobilizes NEDD4-LITAF to recruit ESCRT for membrane repair. Ca^2+^ influx activates dual pathways of membrane repair: ESCRT-III-mediated pore excision: ESCRT-III assembly is initiated by ALG-2/ALIX for membrane extrusion and recycled by VPS4; ASM-dependent endocytosis: Ca²⁺-induced lysosomal exocytosis releases pro-ASM, generating ceramide (Cer) via sphingomyelin (SM) hydrolysis to drive pore internalization. Caspase-7 traverses pores to degrade pro-ASM, augmenting repair efficacy. PMR arises synergistically from pyroptotic pore-driven osmotic swelling and NINJ1 oligomerization, enabling macromolecule release.

Interestingly, independent of Ca²⁺ influx, K⁺ efflux activates the ubiquitin ligase neural precursor cell expressed developmentally down-regulated 4 (NEDD4). This mobilizes lipopolysaccharide-induced tumor necrosis factor-α factor (LITAF), which subsequently reorients to pore-containing endosomes and recruits the ESCRT machinery for membrane repair. [83, 84]. This process internalizes the pore into intraluminal vesicles (ILVs) within multivesicular bodies (MVBs). Instead of excising the pores directly from the plasma membrane, this mechanism ejects the damaged membrane in the form of exosomes [84]. These findings suggest that various membrane repair mechanisms are not independent but may work together to prevent excessive tissue damage and inflammation, ultimately leading to cell survival rather than cell death (Fig. 2).

### Targeting pyroptotic cell membranes in cancer therapy

Plasma membrane damage can trigger the activation and release of inflammatory mediators, leading to pyroptosis and the activation of anti-tumor immune responses. This insight suggests that promoting lipid peroxidation of the cell membrane to damage the plasma membrane may be an effective strategy for combating tumors. A variety of photosensitizers with membrane-anchored properties have been developed to induce lipid peroxidation upon light activation. This process activates caspase-1, which initiates pyroptosis and stimulates T-cell immunity [85–88]. Additionally, cell patches based on freeze-dried hydrogel have been developed recently, which act as calcium chelators. These patches inhibit ESCRT recruitment to the damaged cell membrane, thereby accelerating the process of tumor cell pyroptosis [89].

## Lysosomes

### Inducing factors for LMP

Lysosomes are single-membrane organelles containing various acid hydrolases. Lysosomal enzymes digest cellular compounds in an acidic environment, but some (e.g., cathepsin B (CTSB)、cathepsin S (CTSS)、cathepsin L (CTSL)) remain active at higher pH levels; their leakage into the cytoplasm may activate pyroptosis [90].

LMP, accompanied by the release of cathepsins, particularly CTSB, are key regulatory factors in pyroptosis [11]. LMP can be triggered by a variety of endogenous and exogenous factors. Endogenous triggers include intracellular oxidative stress, fluctuations in Ca²⁺ concentration, and the accumulation of cellular metabolic byproducts [90]. Exogenous factors may include certain drugs, environmental toxins, pathogen invasions, and lysosomal disruptors (e.g., crystalline materials, chemical compounds, nanomaterials, rare earth oxides) [91–94]. These factors induce LMP and subsequent pyroptosis, which may be a new target for cancer treatment.

### Mechanism of LMP-induced pyroptosis

**Fig. 3:**
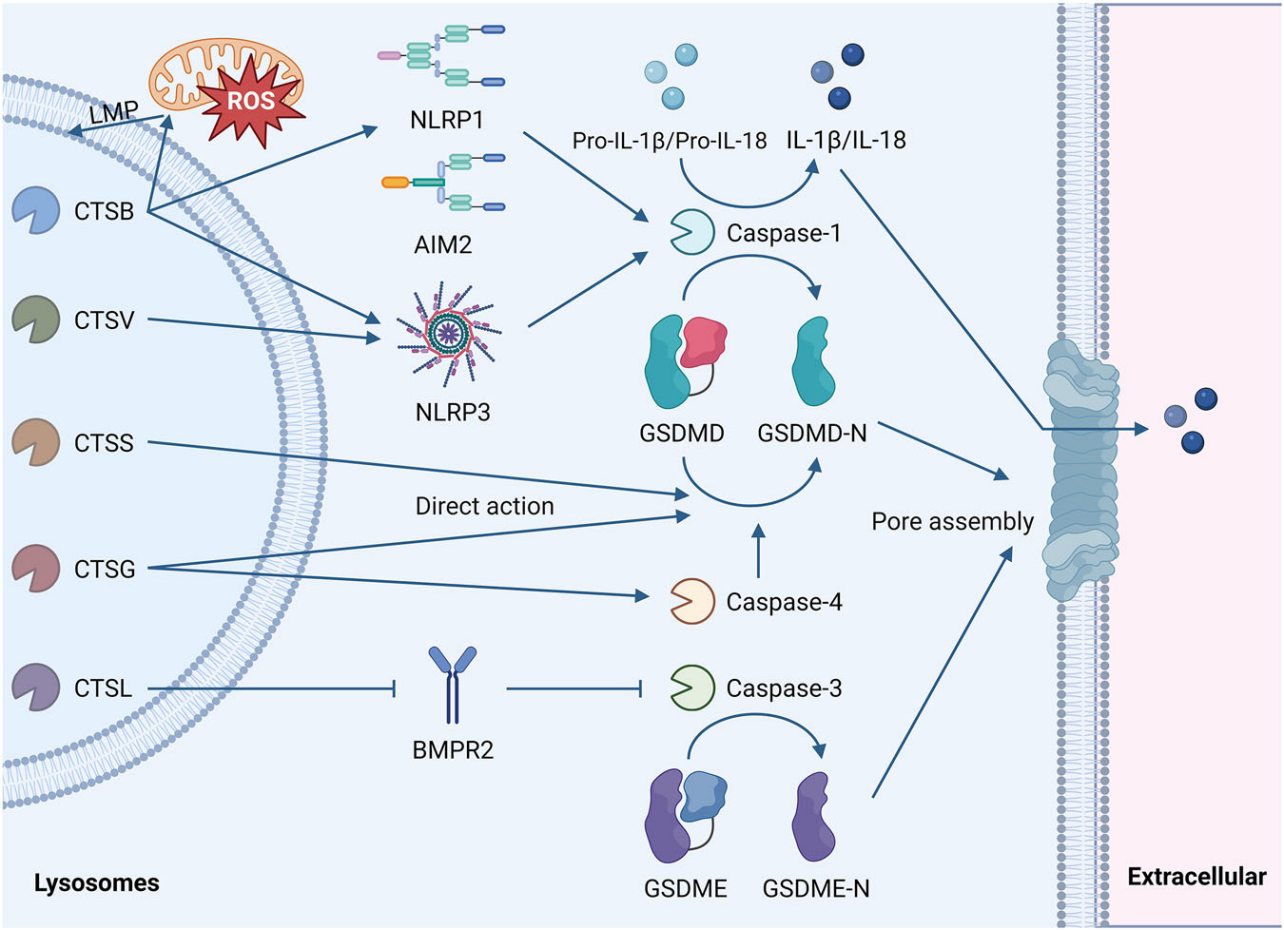
Mechanism of lysosomal instability-induced pyroptosis. Inducing factors, such as ROS, act on lysosomes, leading to lysosomal destabilization and subsequent release of cathepsins from the lysosomal lumen. CTSB and CTSV activate caspase-1 through inflammasome activation, subsequently cleaving GSDMD to generate GSDMD-N. CTSG can cleave GSDMD either by activating caspase-4 or through direct action. CTSS can also directly cleave GSDMD. CTSL promotes GSDME cleavage by degrading BMPR2, thereby relieving its inhibitory effect on caspase-3 activation.

LMP leads to the release of cathepsins, which subsequently cleave GSDM and result in pyroptosis (Fig. 3). Many cathepsins have been confirmed to activate inflammasomes and cleave GSDM, which leads to pyroptosis. Numerous studies have demonstrated that CTSB is associated with pyroptosis via inflammasome activation (NLRP1/NLRP3/AIM2) and caspase-1 [13, 95, 96]. CTSB is known to interact directly with the LRR domain of NLRP3 [97]. In macrophages, CTSB and NLRP3 co-localize in the endoplasmic reticulum region, and their interaction has been detected [98]. These findings suggest that CTSB may bind to the LRR domain of NLRP3 to activate it, thereby providing insights into the molecular interaction mechanism by which CTSB induces pyroptosis in cancer cells. Additionally, cathepsin V (CTSV) mediate pyroptosis through the NLRP3/caspase-1/GSDMD pathway [99].

Some cathepsins have also been demonstrated to induce pyroptosis via the non-canonical pathway. CTSG can induce pyroptosis in two ways: it directly cleaves GSDMD at the Leu274 site to generate GSDMD-N and trigger pyroptosis, and it also activates caspase-4, which then cleaves GSDMD to induce pyroptosis [100, 101]. Besides CTSG, CTSS can also cleave GSDMD independently of caspase-1 [102].

Interestingly, cathepsin L (CTSL) degrades bone morphogenetic protein type II receptor (BMPR2), mediating pyroptosis indirectly. BMPR2 can inhibit pyroptosis-induced cell lysis by suppressing the activation of caspase-3/GSDME signal pathway. However, CTSL is the first identified endogenous lysosomal protease of BMPR2, which can relieve this inhibition [103].

Notably, cathepsins in pyroptosis involve cross-functional interactions, requiring consideration of counterparts [104]. Besides, due to the complexity of regulation induced by cancer-specific factors, future pyroptosis-targeted anti-cancer strategies need to focus on cancer-specific pathways.

### Targeting lysosome to induce pyroptosis in cancer therapy

Lysosomal inhibitors and drugs that induce LMP have been shown to effectively trigger cancer cell pyroptosis. Previous studies have demonstrated that broad-spectrum lysosomal inhibitors (e.g., bafilomycin A1, concanamycin, and archazolide A) and autophagy inhibitors (e.g., antimalarials and their derivatives, chloroquine and hydroxychloroquine) promote lysosomal deacidification, which enhances the susceptibility of lysosomes to LMP induced by anti-cancer agents [105–107]. These inhibitors have been applied in the treatment or therapeutic improvement of various cancers. Also, anti-cancer agents such as Prosapogenin A and the combination of SGI-1027 with everolimus induce pyroptosis in cancer cells through a shared mechanistic pathway centered on inducing LMP and triggering GSDME-dependent pyroptosis [108, 109]. In addition, near-infrared light activation of an antibody-ICG conjugate disrupts lysosomes, enabling the escape of CTSS to directly cleave GSDMD and trigger pyroptosis in cancer cells [102].

Besides, using nanomaterials to induce pyroptosis for cancer therapy has emerged as a popular research area. Homomultivalent Polymeric Nanotraps could trigger lysosomal stress and exert CTSB release, while metal nanoparticles can cause lysosomal damage by particle endocytosis; both of them result in pyroptosis through the NLRP3/caspase-1/GSDMD pathway [110, 111]. Also, Cu-LDH nanoparticles induce cancer pyroptosis by lysosomal rupture-triggered inflammasome/caspase-1/GSDMD pathway and autophagy inhibition-mediated accumulation of pyroptosis-executing GSDMD-N, synergistically amplifying cell death for therapeutic suppression [112]. And the nanomaterials mentioned above have all been tested in vivo. In the future, nanomaterials are set to become a research and application hotspot in cancer therapy based on lysosome-mediated pyroptosis.

## Mitochondria

### Roles of mitochondria in pyroptosis

**Fig. 4:**
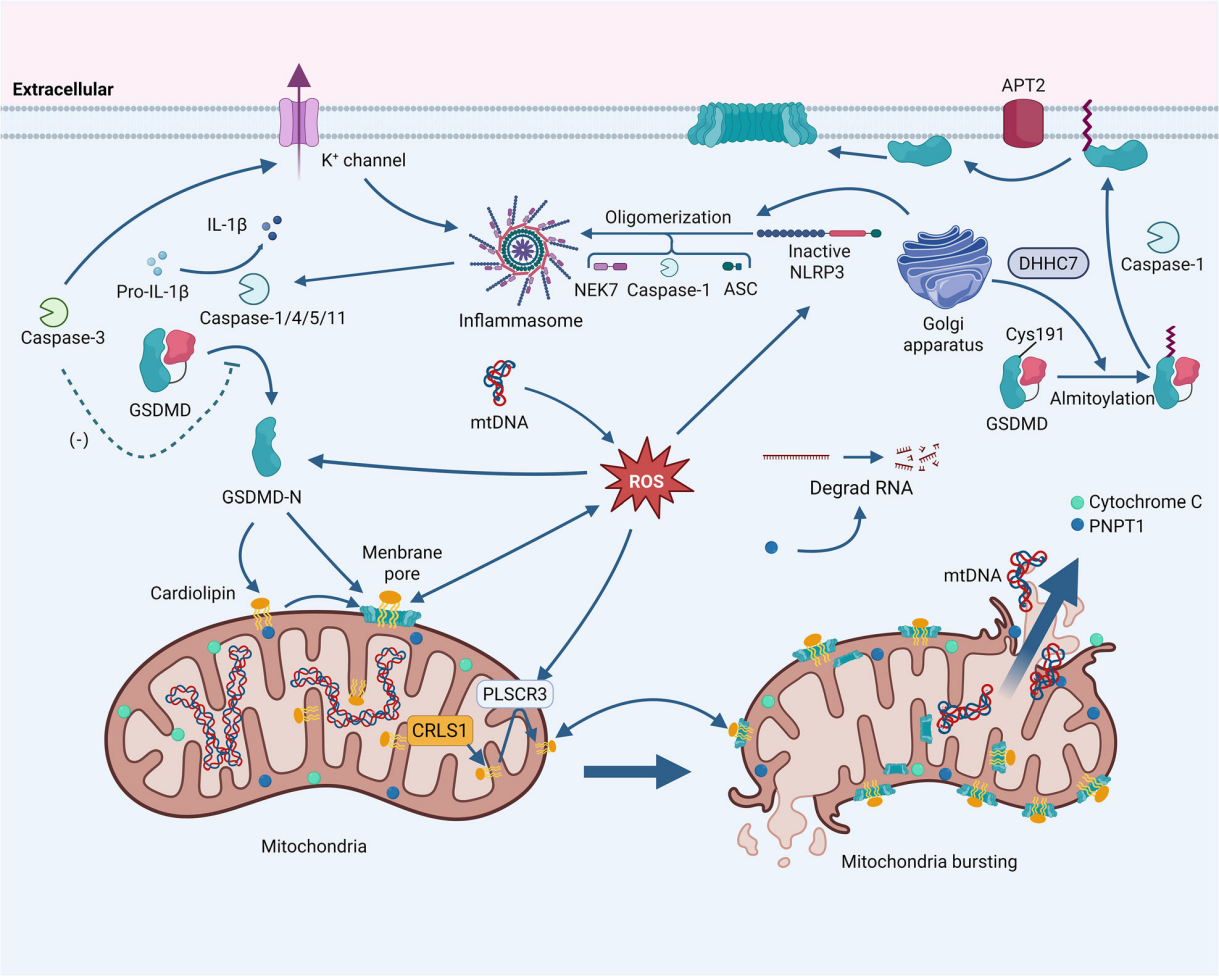
The central role of mitochondria and Golgi Apparatus in orchestrating and amplifying the pyroptotic signaling cascade. GSDMD-N specifically translocates to the mitochondria and binds to cardiolipin, a unique phospholipid on the mitochondrial membrane, to form pores. This initial damage initiates a positive feedback loop where CRLS1 and PLSCR3 work to synthesize and expose more cardiolipin, thereby amplifying the GSDMD-N-mediated pore formation. Sustained assault by GSDMD-N leads to mitochondrial membrane rupture and eventual mitochondrial bursting. This catastrophic event releases various mitochondrial components into the cytoplasm, including mtDNA, which can further activate the inflammasome, and PNPT1, which promotes pyroptosis by degrading RNA. Throughout this process, dysfunctional mitochondria generate a massive burst of ROS, which acts as a central signaling hub to potentiate NLRP3 inflammasome activation, creating a self-reinforcing death cycle. Additionally, the function of GSDMD can be modulated by post-translational modifications, such as palmitoylation at the Golgi apparatus, which is regulated by DHHC7 and APT2. Other caspases, like caspase-3, are also implicated in this network, potentially cleaving GSDM proteins to contribute to pyroptosis.

Mitochondria play a significant role in cell pyroptosis [113, 114]. During this process, mitochondrial dysfunction manifests as a burst of ROS from the respiratory chain complexes, impaired oxidative phosphorylation, and reduced ATP production [115]. TNF-α induces pyroptosis by causing mitochondrial dysfunction and increasing ROS generation, which activates the classical NLRP3/caspase-1/GSDMD-mediated pyroptosis pathway [115–117]. Oxidative stress leads to a decrease in mitochondrial membrane potential, activating the inflammasome and cleaving GSDMD to generate GSDMD-N [5, 118]. This fragment binds preferentially to cardiolipin, a phospholipid on the outer mitochondrial membrane, forming pores in the membrane. This pore formation leads to the release of molecules such as mtDNA and cytochrome c. The released cytochrome c triggers a caspase cascade, while the exposure of cardiolipin creates a positive feedback loop through its synthesis by cardiolipin synthase 1 (CRLS1) and its translocation to the outer membrane mediated by phospholipid scramblase 3 (PLSCR3), further amplifying the pyroptotic signal. [115, 119]. Concurrently, the cytoplasmic release of polynucleotide nucleotidyltransferase 1 (PNPT1) from mitochondria causes mRNA degradation, inhibiting protein synthesis and thereby promoting cell death [118] (Fig. 4).

### Morphological changes of mitochondria during pyroptosis

Normal mitochondria possess a double-membrane structure, with the inner membrane folded into cristae to maintain oxidative phosphorylation function [116, 120]. During pyroptosis, mitochondria undergo rapid and dramatic morphological changes: initially, they quickly become rounded and shorten by approximately half, accompanied by autophagosomes clearing damaged organelles. In later stages, about 60% of mitochondria exhibit loss or disorientation of cristae, membrane rupture, and an overall reduction in number [115, 121]. The GSDMD-N can directly damage both mitochondrial membranes, increasing permeability and causing rupture. This structural damage marks a point of no return in the cell’s irreversible progression toward death [115].

### Targeting mitochondria for tumor pyroptosis intervention

Targeting and regulating mitochondria can effectively intervene in tumor pyroptosis. Nanomedicines, such as mitochondria-targeted melatonin and tetraethylammonium enyne-based compound, induce pyroptosis by delivering ROS inducers to a specific target, thereby triggering an ROS burst and GSDME cleavage [122]. A photosensitizer named Th-M can target mitochondria and, upon white light irradiation, trigger mitochondrial dysfunction. This leads to the cleavage of caspase-3 and GSDME, thereby inducing pyroptosis in tongue squamous cell carcinoma cells. [123, 124]. Additionally, liver X receptor agonists activate the caspase-4/apoptotic protease-activating factor 1 (APAF-1) pyroptosome by altering the integrity of the outer mitochondrial membrane, inducing GSDME-mediated pyroptosis in tumor cells and offering a new direction for anti-tumor therapy [125]. And the nanomaterials mentioned above have all been tested in vivo.

## Golgi Apparatus

### Roles of the Golgi apparatus in pyroptosis

The Golgi apparatus, as a core regulator of the endomembrane system, plays a critical role in pyroptosis through multidimensional mechanisms, including dynamic protein modification, secretory functions, and structural responses [126]. In terms of dynamic protein modification, the Golgi-localized palmitoyltransferase DHHC domain-containing palmitoyltransferase 7 (DHHC7) catalyzes the palmitoylation of GSDMD at the Cys191 site. This enhances its interaction efficiency with caspases and promotes the cleavage of the GSDMD-N and its translocation to the plasma membrane [127]. Conversely, the acylthioesterase acyl protein thioesterase 2 (APT2) on the plasma membrane releases the Cys191 site through depalmitoylation, allowing its oxidative modification under the action of ROS, ultimately driving GSDMD-N oligomerization and membrane pore formation [16]. Furthermore, studies have found that cleavage-deficient GSDMD (D275A) also undergoes palmitoylation upon inflammasome stimulation or treatment with ROS activators and induces pyroptosis, although less efficiently than palmitoylated GSDMD-N. This implies that palmitoyltransferase-catalyzed GSDMD palmitoylation inducing pyroptosis is a relatively independent pathway [17] (Fig. 4).

### Impact of Golgi apparatus structural damage on the activation of pyroptotic pathways

Under normal physiological conditions, the Golgi apparatus is typically composed of a stack of flattened, membrane-bound sacs (called “cisternae”), responsible for protein modification, sorting, and transport. However, under pyroptotic or stress conditions (e.g., photodynamic therapy or oxidative stress), the Golgi apparatus structure undergoes diffusion or fragmentation, leading to its functional disruption. For example, Golgi damage induced by photodynamic therapy can significantly promote the NLRP3/caspase-1/GSDMD pathway to release pro-inflammatory cytokines IL-1β and IL-18 and induce pyroptosis [128]. Furthermore, the Golgi disruptor Golgicide A can induce pyroptosis in lung cancer stem cells by affecting the formation of the trans-Golgi network [129].

## Endoplasmic Reticulum

### Mechanisms linking endoplasmic reticulum stress to pyroptosis

**Fig. 5:**
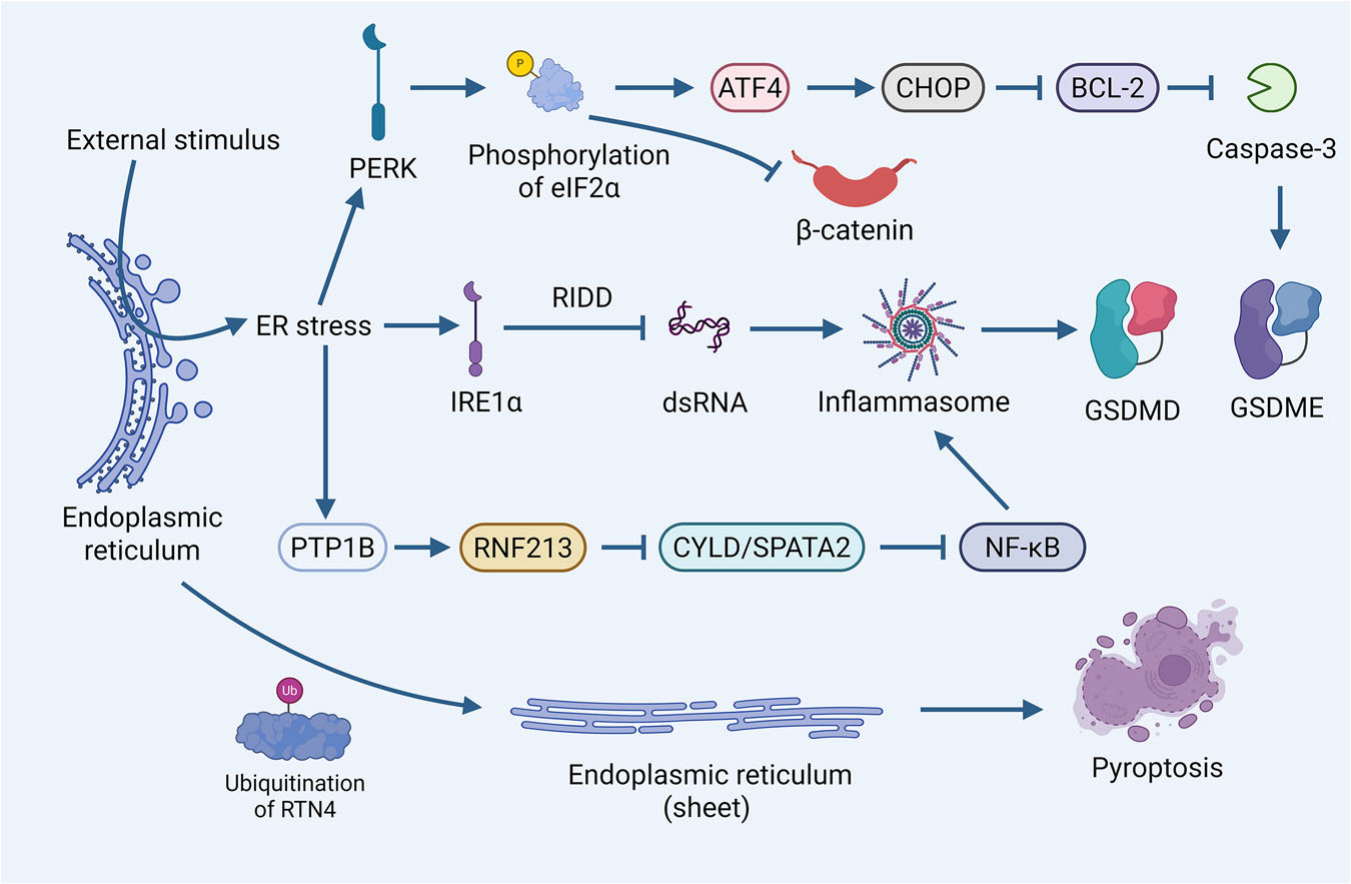
The role of endoplasmic reticulum stress, UPR signaling, and structural dynamics in the regulation of pyroptosis ER stress regulates cell pyroptosis through multiple branches of the UPR. Inhibition of the RNase activity of IRE1α can lead to the accumulation of dsRNA, which in turn enhances the activation of the NLRP3 inflammasome, inducing pyroptosis. The PERK branch, through the phosphorylation of eIF2α, promotes ATF4 translation and CHOP expression. CHOP then triggers pyroptosis by inhibiting Bcl-2 and activating caspase-3, which cleaves GSDME. Additionally, ER stress induced by factors such as hypoxia can upregulate PTP1B. PTP1B, by regulating RNF213 activity, promotes the degradation of CYLD/SPATA2. This activates the NF-κB pathway, upregulating the expression of NLRP3 inflammasome components, and ultimately activating the NLRP3 inflammasome to cleave GSDMD, causing pyroptosis. The structural dynamics of the ER are also crucial: the natural compound α-MG or UBR5-mediated ubiquitination and degradation of RTN4 causes the collapse of the ER’s tubular structure and promotes ER-plasma membrane fusion to form “bubble-like” structures, directly inducing pyroptosis. This process can be accompanied by the translocation of ER marker proteins like Calnexin to pyroptotic bodies, further exacerbating mitochondrial Ca²⁺ overload and ROS bursts, creating a vicious amplification loop.

ER is pivotal for protein folding and lipid synthesis. Accumulation of misfolded/unfolded proteins triggers ER stress, a condition common in tumor cells due to their rapid proliferation and metabolic aberrations. Cells counteract this by activating UPR, mediated by IRE1α, PERK, and ATF4 sensors [130] (Fig. 5).

Among these, the regulated IRE1-dependent decay (RIDD) activity of IRE1α can degrade mRNA, including mRNA potentially involved in regulating pyroptosis. Inhibition of IRE1α’s RNase activity leads to dsRNA accumulation, thereby enhancing NLRP3 inflammasome activation and pyroptosis [131]. PERK, by phosphorylating eIF2α, promotes the translation of transcription factor ATF4, which in turn induces the expression of the pro-apoptotic factor CHOP. CHOP can directly or indirectly inhibit the anti-apoptotic protein Bcl-2 and activate caspase-3 to cleave GSDME, releasing pore-forming fragments that trigger pyroptosis. Furthermore, hypoxia-induced ER stress elevates protein levels of protein tyrosine phosphatase 1B (PTP1B) in cells [132]. PTP1B, by regulating the activity of RNF213, induces the degradation of the major NF-κB regulator cylindromatosis and spermatogenesis associated 2 (CYLD/SPATA2). The reduction of CYLD/SPATA2 leads to the activation of the NF-κB pathway, promoting the expression of NLRP3 inflammasome components. Ultimately, under appropriate stimulation, the NLRP3 inflammasome is activated and triggers GSDMD cleavage, leading to pyroptosis (Fig. 5).

### Morphological changes of the endoplasmic reticulum in pyroptosis

The normal ER is a dynamic network of tubules and sheets. The natural compound α-Mangostin (α-MG) induces ubiquitination and degradation of Reticulon-4 (RTN4) via UBR5. This causes ER tubular structures to collapse into sheets and promotes ER-plasma membrane fusion, facilitating the formation of “bubble” structures that contribute to pyroptosis [133].

### ER-targeted strategies to modulate pyroptosis for optimizing anti-tumor therapy

Targeting key molecules or pathways of the ER can modulate the pyroptosis process to optimize anti-tumor efficacy. In colon tumors, knockdown of IRE1α activates the PERK/eIF2α signaling pathway, leading to the inhibition of β-catenin production [134]. A novel short peptide, D-FFxFFs, can specifically trigger ER-phagy, causing ER Ca^2+^ release and a surge in mitochondrial Ca^2+^ levels, thereby inducing GSDMD-mediated pyroptosis and activating immune responses against tumor cells, which has been tested in vivo. [135]. CS-HAP@KAE NPs can precisely reach tumor cells and induce Ca^2+^ overload, subsequently causing ER stress, enhancing the expression of the STING/IRF3 signaling pathway, and promoting the NLRP3/caspase-1/GSDMD pathway, which has just been tested in vitro [136]. Conversely, STING depletion specifically triggers abnormal activation of the PERK/eIF2α/ATF4/CHOP axis in renal cell carcinoma cells, leading to reduced protein synthesis and ultimately resulting in caspase-8 cleavage-mediated GSDMD-dependent pyroptosis. Therefore, targeted inhibition of STING can induce GSDMD-dependent pyroptosis and enhance anti-tumor immunity in renal cell carcinoma [137]. These strategies, by intervening in ER dynamics and signaling pathways, offer new directions for precision tumor therapy.

## Extracellular Vesicles (Evs)

### EVs in pyroptosis

Pyroptosis is also characterized by vesicle shedding. For example, with a diameter of generally 40–150 nm [138], exosomes play a significant role in paracrine signaling as EVs, protecting and transferring mRNA and miRNA to recipient cells, facilitating RNA-mediated intercellular communication [139]. During pyroptosis, exosomal changes are primarily reflected in the upregulation of molecules and proteins they carry, which are subsequently released into the extracellular environment, influencing surrounding cells. Below are key points regarding exosomal changes during pyroptosis:

Pyroptotic EVs contain active caspase-1 [140], GSDMD [141], DAMPs [54, 63, 142], as well as NLRP1 [66]. They are responsible for releasing interleukin IL-1β and IL-18. In pyroptotic EVs, caspase-1 appears to be dispensable for bystander killing, as EV-caspase-1 still requires GSDMD in recipient cells to induce cell death [141]. When PMR occurs during pyroptosis, intracellular proteins such as HMGB1 and LDH (a standard PMR marker) are released into exosomes. PD-L1 may also appear in exosomes, as evidenced in breast cancer [143], melanoma [144], prostate cancer [145], and lung cancer [146]. As pyroptosis occurs, cells release a large number of exosomes into the surrounding environment, thereby transmitting pyroptotic signals between cells.

Exosomes not only act as signaling mediators but also interact with recipient cells through surface molecules, regulating immune responses and other biological processes. For example, hepatocyte-derived exosomes containing HMGB1 deliver extracellular LPS to the cytoplasm and mediate pyroptosis [147]. The recognition of cytoplasmic LPS by caspase-11 leads to pyroptosis and the secretion of inflammatory mediators [148].

The alterations in EVs during pyroptosis provide a blueprint for further investigating their mechanisms in inducing pyroptosis.

### Mechanism of EVs inducing pyroptosis

Pyroptotic cells release EVs that transfer GSDMD pores and mtDNA to recipient cells, triggering inflammation and cytolysis. Specific miRNAs (e.g., miR-196-5p) further promote disease progression associated with pyroptosis.

Exosomes released by pyroptotic cells contain GSDMD pores, which can be transferred to the membranes of bystander cells, propagating lytic cell death and promoting inflammation, supported by the detection of GSDMD-N in pyroptotic exosomes released from iBMDM-iGSDMD-N cells via immunoblotting [141]. Exosomes may regulate intracellular communication and contribute to bystander cell death [149–151]. For instance, isolated pyroptotic EVs can mediate the death of HeLa cells. Pre-formed GSDMD pores are released from apoptotic cells onto exosomes and integrate into the plasma membranes of bystander cells, triggering their extracellular lysis. GSDMD pores released on exosomes remain transplantable and cell-lytic [141]. Ras GTPase-activating-like protein IQGAP1 functions as an adapter, bridging GSDMD to the ESCRT machinery to promote the biogenesis of pro-IL-1β-containing exosomes in response to NLRP3 inflammasome activation [152].

GSDMD also promotes the release of mtDNA. Pyroptotic cells secrete mtDNA encapsulated within exosomes. Activation of caspase-1 leads to mtDNA leakage from mitochondria into the cytoplasm via GSDMD. Caspase-1 also induces intraluminal membrane vesicle formation, allowing cellular mtDNA to be taken up and secreted as exosomes [153].

Certain miRNAs within exosomes can also promote pyroptosis. MiR-196-5p is highly expressed in exosomes secreted by tumor cells, which negatively targets inhibitor of growth 5 (ING5), promoting tumor cell growth. Cancer-derived exosomes promote T cell pyroptosis, further exacerbating cancer progression. Exogenous miR-196b-5p can induce T cell pyroptosis through suppressing ING5 levels, promoting tumor growth and accelerating NSCLC progression [154].

Through crosstalk among multiple organelles, EVs mediate pyroptosis, offering insights into the conversion of intracellular signals into extracellular signaling processes.

### EVs therapeutics for pyroptosis

Given the fact that exosomes promote pyroptosis through the changes of their content, exosomes are now utilized as carriers, with rational modifications for therapeutic interventions. The use of exosomes in these applications can be termed exosomal therapeutics.

Exosomes can serve as carriers to regulate pyroptosis, metabolic disorders, microenvironments, and cellular homeostasis, thereby achieving therapeutic effects. Cancer cell-derived exosomes can encapsulate an antagonist of the extracellular enzyme CD39 (POM1) and an AMPK agonist (metformin), serving as tumor-targeting nanocarriers [155]. Moreover, clinical drugs and drug conjugates are also viable [156, 157]. Additionally, emerging modification strategies such as hydrogels and nanomaterials highlight the broad prospects of EV-mediated pyroptosis therapy.

## Cytoskeleton

### Structural mechanism of the cytoskeleton inducing pyroptosis

The cytoskeleton is composed of microfilaments, microtubules, and intermediate filaments. Given that inflammasome assembly requires the convergence of multiple components into a single inflammasome speck, it can be inferred that the cytoskeleton is involved in this process to some extent [158].

The microtubule-organizing center (MTOC) is the site responsible for the assembly of the NLRP3 and pyrin-mediated inflammasomes, caspase activation, and IL-1β processing [159]. This process is catalyzed and regulated by various enzymes, including but not limited to microtubule affinity-regulating kinase 4 (MARK4) [160], histone deacetylase 6 (HDAC6) [159], NIMA-related kinase 7 (NEK7) [161], and phosphorylated ATP-citrate lyase (ACLY) [162].

In THP-1 cells and bone marrow-derived macrophages (BMDMs), NLRP3 can bind to MARK4, which drives NLRP3 to the MTOC, resulting in the formation of a large inflammasome speck complex within a single cell, thereby promoting optimal inflammasome activation [160].

One of the transporters of inflammasome sensors is the dynein adapter HDAC6 [159]. In immortalized BMDMs and THP-1 cells, HDAC6 is essential for the transport of NLRP3 and pyrin to the MTOC, where they each form a single inflammasome complex. Microtubules regulate the intracellular sorting of NLRP3, mediate NLRP3 inflammasome assembly, and microtubule disruption attenuates NLRP3 inflammasome activation [163, 164]. Here’s a centrosomal protein called NEK7 that plays a significant role in regulating NLRP3 activity [161, 165]. Before the localization of NLRP3 at the MTOC, LATS1/2, pre-recruited to the MTOC, phosphorylate NLRP3, further promoting its interaction with NEK7, ultimately achieving full activation of NLRP3 [166].

In pyroptosis, ACLY-mediated microtubule dynamics are involved in the progression of inflammation. Phosphorylated ACLY, by affecting tubulin acetylation, targets the microtubule cytoskeleton, promoting NLRP3 inflammasome activation and pyroptosis [162].

### Critical cytoskeletal components involved during pyroptosis

During pyroptosis, crucial ingredients of cytoskeleton are involved. Septin promotes pyroptosis by regulating GSDMD cleavage and NINJ1 expression, while L-plastin (LPL) enhances NLRP3 assembly. Additionally, actin and intermediate filaments play regulatory roles in pyroptosis.

One of the cytoskeletal components, septin filaments, assemble on the plasma membrane and regulate phosphoinositide levels, mitochondrial dynamics and NINJ1 expression, thereby modulating PMR and promoting macrophage pyroptosis [167]. Besides, actin-bundling protein LPL significantly enhances NLRP3 assembly by contributing to the NLRP3-ASC-caspase-1 complex formation [168]. Meanwhile, inactivating actin-severing protein cofilin, which is involved in the actin depolymerization, inhibits cisplatin-induced pyroptosis [169]. Targeting cytoskeletal components may represent a novel therapeutic approach for pyroptosis modulation.

### Destructive mechanism of the cytoskeleton during pyroptosis

Pyroptosis induces extensive cytoskeletal disruption. Calpain-mediated vimentin cleavage increases cellular fragility, while intracellular tension regulates cell swelling and membrane blebbing.

The hallmark features of pyroptosis include cell swelling, membrane blebbing, and the release of inflammatory cytoplasmic contents [170, 171]. The cytoskeleton plays a crucial role in these processes. These events were once thought to be driven by colloidal osmotic lysis. However, pyroptotic cells do not actually undergo lysis in vitro but lose mechanical elasticity. In fact, calpain cleaves vimentin, leading to the loss of intermediate filaments, making cells fragile and prone to rupture under external stress, such as shear stress or compression [172]. When cells swell and rupture due to factors other than osmotic pressure, in addition to the loss of intermediate filaments, other cytoskeletal networks, such as microtubules, actin, and the nuclear lamina, are also disrupted during pyroptosis [171], which is confirmed by cellular analysis, and the sensitivity to rupture is associated with calpain-dependent vimentin cleavage and intermediate filament loss.

**Fig. 6:**
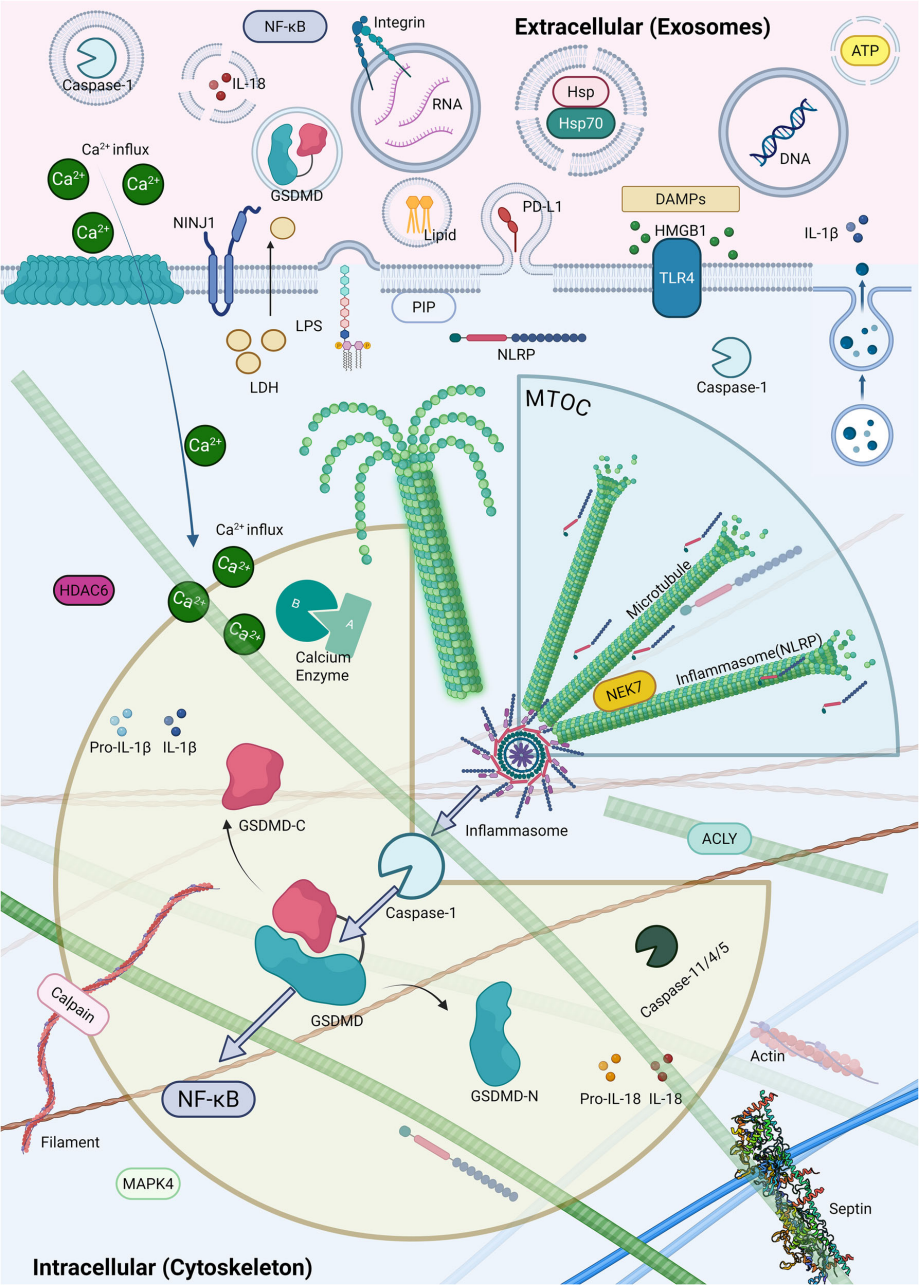
The role of cytoskeleton and exosomes in pyroptosis. In pyroptosis, various components of the cytoskeleton are damaged under mechanical stress and osmotic pressure. During this process, physiological events such as Ca²⁺ influx and calpain-mediated cleavage of intermediate filaments occur. The MTOC plays a crucial role in assembling the inflammasome and activating its maximum activity, a process regulated and catalyzed by various enzymes, including MAPK4, HDAC6, NEK7, and ACLY. Successful assembly of the inflammasome further activates caspase-1, which cleaves GSDMD, thereby activating the pyroptosis through NF-κB pathway. As part of the cytoskeleton, septin is capable of mediating pyroptosis characterized by PMR by regulating the activity of GSDMD and NINJ1. Exosomes play a key role in the spread of pyroptosis, which is closely related to changes in their contents. Pyroptotic exosomes carry GSDMD, transferring it to neighboring cells and causing a wider range of pyroptosis; they can also carry related RNA and DNA, regulating various inflammatory pathways to trigger downstream inflammatory responses. The cargo of exosomes during this interaction includes but is not limited to DAMPs (such as HMGB1), caspase-1, IL-18, IL-1β, LPS, ATP, and PD-L1.

Additionally, intracellular tension plays a role in pyroptosis. Protein nanoparticle-induced osmotic pressure (PN-OP) is involved in the swelling and membrane blebbing of pyroptotic astrocytes and is closely related to inflammasome formation and cytoskeletal depolymerization. It has been observed that the activation of GSDMD, through inflammasome-induced upregulation of osmotic pressure and increased Ca²⁺, participates in the modification of typical pyroptotic features. Inflammasomes, acting as protein nanoparticles, are involved in the upregulation of PN-OP and control the characteristic features of pyroptotic astrocytes [170]. Therefore, to draw a conclusion, targeting the cytoskeleton to disrupt tumor cells demonstrates theoretical feasibility as a potential therapeutic strategy (Fig. 6).

## Membraneless Organelles

As membraneless organelle networks, stress granules (SGs), centrosomes, and nuclear speckles function as dynamic hubs that spatiotemporally regulate inflammasome activity and pyroptosis.SGs attenuate pyroptosis by sequestering the RNA helicase DDX3X, which otherwise promotes NLRP3 activation [173–175]. Centrosomes provide a spatial platform for NEK7-dependent NLRP3 inflammasome assembly through microtubule-mediated trafficking [176–178]. In contrast, nuclear speckles promote tumor immune evasion through the overexpression of serine proteinase inhibitor, clade B, member 6B (SERPINB6B), driven by the long non-coding RNA, metastasis-associated lung adenocarcinoma transcript 1(MALAT1); this inhibits GSDMD cleavage and pore formation. [179]. Targeting these organelles with SG-disassembly agents, NEK7 stabilizers, or MALAT1-directed antisense oligonucleotides, therefore, represents a compartment-specific therapeutic strategy to restore or enhance tumor pyroptosis (Table 2).

**Table 2:** Regulatory roles of membraneless organelles in pyroptosis and therapeutic implications.

Organelles	Key Elements	Mechanism of Action	Therapeutic Targets	Ref.
Stress Granules	DDX3X	Sequesters DDX3X to limit NLRP3 inflammasome activation	SG disassembly agents; DDX3X mutants	[160–162]
Centrosome	NEK7 kinase	Microtubule-mediated NLRP3 trafficking for inflammasome assembly	NEK7 stabilizers; Recombinant NEK7 delivery	[178–180]
Nuclear Speckles	MALAT1/SERPINB6B	SERPINB6B inhibits GSDMD cleavage via MALAT1 upregulation	MALAT1 ASOs; SERPINB6B CRISPRi	[181]

## Crosstalk of organellar networks in pyroptotic signaling

**Fig. 7:**
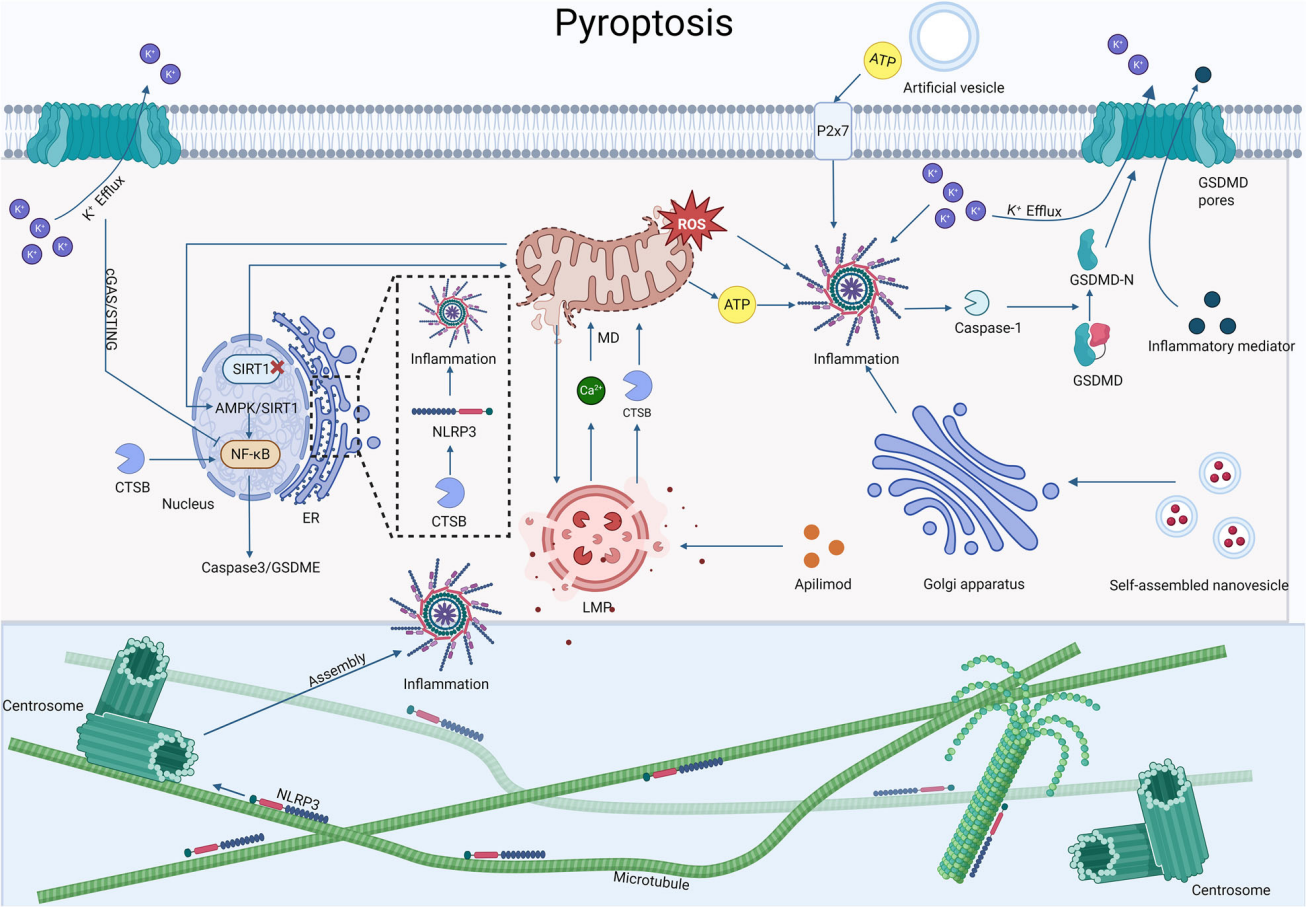
Organelle networks coordinating pyroptosis and therapeutic targets. Mitochondrial-lysosomal crosstalk drives pyroptosis through reciprocal ROS/Ca²⁺ signaling, where mitochondrial damage activates NLRP3 inflammasomes and lysosomal Ca²⁺ efflux exacerbates mitochondrial dysfunction, while mitochondrial ROS trigger lysosomal permeabilization to release CTSB, amplifying ROS production [12, 13]. CTSB activates NF-κB to further enhance inflammatory signaling and interacts with NLRP3 at the ER to promote inflammasome assembly [98, 181]. Nuclear-mitochondrial coordination modulates pyroptotic sensitivity via SIRT1-mediated mitochondrial stability and AMPK/SIRT1/NF-κB-driven caspase-3/GSDME activation [26, 180]. Microtubule-associated proteins direct NLRP3 transport to centrosomes for inflammasome assembly [158–160]. Golgi apparatus-targeted photodynamic therapy via nanovesicles activates NLRP3 to induce pyroptosis.[128] K^+^ efflux primes inflammasomes while suppressing cGAS-STING/NF-κB immunity [57, 58], and ATP-loaded vesicles engage urinergic 2 X receptor 7 (P2X7) to selectively trigger tumor pyroptosis [155, 182, 183].

The interplay among nucleus, mitochondria, lysosomes, ER, Golgi apparatus, and cytoskeletal structures establishes a multilayered framework for pyroptosis execution. Mitochondria serve as the central hub, releasing ROS and ATP to amplify inflammasome activity, while lysosomal crosstalk reinforces this process through CTSB release [12, 13]. ER platforms and nuclear-mitochondrial coordination further tune sensitivity, with metabolic stress (SIRT1/AMPK/NF-κB) linking mitochondrial dysfunction to caspase-3/GSDME activation [26, 98, 180, 181]. The cytoskeleton spatially organizes inflammasome assembly, and Golgi-targeted photodynamic therapy has emerged as an innovative trigger of pyroptosis [128, 158–160]. Downstream, pyroptotic pores drive cytokine release, with K^+^ efflux and extracellular ATP shaping immune surveillance [57, 58, 182, 183]. Collectively, these organelle interactions highlight targetable nodes that could be exploited to induce tumor pyroptosis and overcome therapeutic resistance (Fig. 7).

## Conclusion and future outlook

This review has systematically synthesized the critical roles of organelles in pyroptosis, establishing an organelle-centric paradigm that moves beyond a linear, pathway-centric view. This holistic perspective not only clarifies the intricate cellular processes underlying pyroptosis but also unveils a spectrum of novel intracellular targets for therapeutic intervention, particularly in cancer. By framing pyroptosis as a cascade of subcellular disasters, we provide a foundational framework for future research and clinical translation. Compared to existing literature, which often delves deeply into specific pathways in isolation, the primary significance of our work lies in integrating these pathways into the spatiotemporal context of organelle dynamics. For instance, while numerous studies have documented mitochondrial dysfunction in pyroptosis, our synthesis positions it as a pivotal amplifier that can dictate the scale and immunogenicity of cell death, a concept less emphasized in pathway-specific reports. What’s more, this review specifically highlights NINJ1 as an active executor of membrane rupture and introduces counter-acting repair mechanisms (ESCRT, ASM) that determine the kinetics and outcome of cell death. This integrated view is crucial for developing sophisticated multi-target strategies.

Looking ahead, several frontiers promise to deepen our understanding. To begin with, the exploration of novel organelles like migrasomes is of paramount significance. This review has charted the known organelle landscape; future work may integrate emerging players. Migrasomes [184] that mediate cellular contents exchange including cytosolic components [185], mRNAs and proteins [186], treatment of damaged organelles [187], and intercellular communication among cells, potentially secreted from tumor cells featuring metastasis, which provides new insights into its roles in tumor prognosis concerning pyroptosis and immunotherapy. Given the migratory nature of migrasomes, it is postulated that they may share exosomes’ capacity to transport GSDMD, a key effector of pyroptosis, potentially serving as an unconventional vehicle for transmitting pyroptotic signals. In tumors with metastatic traits, the overexpression of migrasomes may facilitate the expulsion of pyroptotic factors, thereby accelerating tumor metastasis and enhancing tumor survival mechanisms. As a new target for disease treatment, blocking the intrinsic migration promoting effect of migrasomes through nanoparticles may be a new strategy for anti-metastasis nanotherapy [188]. Another pivotal direction involves determining whether pyroptosis-induced organellar alterations are consistent across different tumor cell types, such as lymphocytes versus solid tumor cells. Systematically delineating these discrepancies is crucial for identifying precise and safe therapeutic targets with cell-type specificity.

**Fig. 8:**
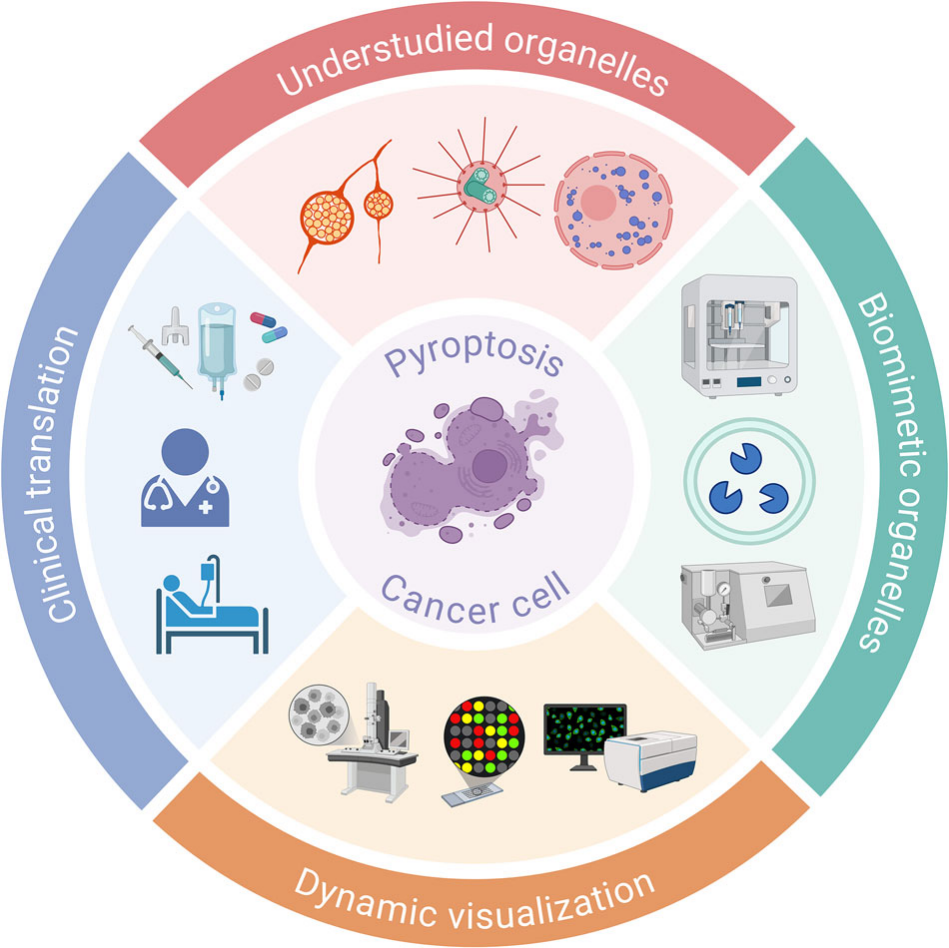
Core research directions. This schematic is divided into four modules illustrating core research directions for inducing cancer cell pyroptosis through organelle-targeting strategies.

Technological innovation from visualization tools to biomimetic therapies will be a key driver for the clinical translation in this field. Defining pyroptosis severity by staging organelle damage, using tools like STED microscopy and organelle-specific fluorescent probes, could transform it from a binary assessment into a quantifiable continuum, providing much-needed biomarkers for drug development. Besides, the translational potential of organelle-level regulation is being unlocked by biomimetic technologies. Artificial exosomes mimicking natural organelles for targeted delivery of pyroptosis modulators represent a promising application. However, in contrast to the robust literature on drug-loaded liposomes, significant challenges in yield, stability, and safety of these sophisticated biomimetics remain and must be addressed through interdisciplinary collaboration. Furthermore, employing advanced microscopy to resolve the ultrastructural details of organelle remodeling in pyroptosis will be imperative to move from schematic models to a mechanistic, high-resolution understanding (Fig. 8).
